# An internet-based educational intervention for mothers targeting preschoolers’ weight management promotion (PWMP): a pilot study

**DOI:** 10.1186/s12889-022-14543-5

**Published:** 2022-11-29

**Authors:** Fazlollah Ghofranipour, Najmeh Hamzavi Zarghani, Eesa Mohammadi, Ali Asghar Haeri Mehrizi, Mahmoud Tavousi, Marieke De Craemer, Greet Cardon

**Affiliations:** 1 Department of Health Education and Health Promotion, Faculty of Medical Science, Tarbiat Modares University, Tehran, Iran; 2 Department of Nursing, Faculty of Medical Science, Tarbiat Modares University, Tehran, Iran; 3 Health Metrics Research Center, Iranian Institute for Health Sciences Research, ACECR, Tehran, Iran; 4 Department of Rehabilitation Sciences, Ghent University, Ghent, Belgium; 5 Department of Movement and Sports Sciences, Ghent University, Ghent, Belgium

**Keywords:** Educational intervention, Mothers, PRECEDE-PROCEED Model, Preschool children, Pilot study, Self-efficacy, Obesity

## Abstract

**Background:**

The prevalence of overweight and obesity among children has raised public health concerns. This study aimed to design and evaluate a behaviour change intervention program to promote weight management among Tehranian preschoolers.

**Methods:**

The PRECEDE-PROCEED model is one of the most popular models in health education used to develop and evaluate most educational interventions. In this one-group pre and post-pilot study, 13 mothers of preschoolers were recruited from preschools in Tehran (the capital of Iran), in August 2020. Mothers received a six-week educational intervention, including text messages and educational videos via WhatsApp, to increase their self-efficacy to overcome barriers changing their children’s lifestyle. Mothers reported preschoolers’ height and weight to assess Body Mass Index and filled out the Food Frequency Questionnaire, the Persian version of the children's health-related quality of life questionnaire, and demographic features. The “Children’s physical activity and sedentary behaviors checklist,” newly designed by the researchers, was also filled out by mothers. These behaviors were measured according to the minutes that children were involved in these activities in a day, and the days they spent in a week for them. All variables were measured at baseline, immediately after the intervention and three months later. Data analysis was performed using SPSS IBM statistics version 22. Friedman test was used to evaluate changes over time.

**Results:**

The findings demonstrated that the mean BMI z-score stayed steady between baseline, immediately after the intervention and after three months (*P* = 0.60). Besides, after three months, the intervention programme led to a decrease in soft drink consumption (*P* = 0.001), and an increase in parental perception of their child’s general health (*P* = 0.05), the parental concern regarding their child’s emotional and physical health (*P* = 0.002) and minutes of physical activity per day (*P* = 0.02). However, fruit intake decreased (*P* = 0.01), and simple sugar, such as cube, increased (*P* = 0.03).

**Conclusion:**

Results from this study are promising but should be interpreted with caution and should be replicated on a larger scale and compared with a control group to evaluate whether effects are maintained in a larger sample.

## Introduction

The prevalence of obesity and overweight during childhood has dramatically increased during the last decades [1, 2]. World Health Organization (WHO) reported that 39 million children under the age of five were overweight or obese, in 2020 [3]. Moreover, among preschoolers in Tehran (the capital of Iran), the year 2011, prevalence rates of overweight and obesity were 7.4% and 4.7% in boys and 11.5% and 11.0% in girls, respectively [4]. The first nationwide survey reported that 17.0% of Iranian preschoolers were living with an overweight or obesity and 12.7% were living with underweight [5]. Additionally, obesity tracks throughout the life course, which means that the risk of obesity during adolescence and adulthood is higher in children under six being obese compared to normal-weight children [6]. Besides, obesity and overweight in childhood may be an indicator of an unhealthy lifestyle and can result in non-communicable diseases, such as diabetes, cancers and cardio-vascular diseases in adulthood [7, 8]. Moreover, psychological disorders including depression, low self-esteem, body image concerns, and also educational progress and quality of life during adulthood can be influenced by overweight in early childhood [8, 9].

Weight management programs, including healthy nutrition, being physically active and reducing sedentary behaviors, are the most important strategies to improve weight control and children’s lifestyle during early childhood [10, 11]. Additionally, behavior change interventions have shown to be effective in obesity prevention and weight management promotion during later childhood and the life course [12, 13]. In this regard, many behavior change interventions have been already performed on behavior change factors (e.g., parents, teachers) and various environments (e.g., preschool, home) by targeting both diet and physical activity behaviours to prevent children’s overweight and obesity [14–18].

Practical guidelines developed by the American Medical Association that focus on reducing obesity among children aged 2–18 years are mainly based on research conducted in older children and adolescents [19]. Additionally, most related studies investigating weight status in Iranian preschoolers have mostly focused on the prevalence of overweight and obesity and also on the risk factors of obesity in children, such as demographic variables, high level of screen time among children and parental obesity [20–23].

The best method for designing and evaluating behavior change interventions may be using behavior change theories and models which the latter are theories in their early stages[24]. The results of a systematic review showed that behavioral models and conceptual frameworks used in childhood obesity prevention included the theory of planned behavior (TPB), health belief model (HBM) and social cognitive theory (SCT) [25]. Literature showed that the SCT was mostly used [25]. Besides, the interventions considered increasing self-efficacy in parents as the key construct of behavior change in SCT, which refers to belief in one's ability to organize and to produce intended achievements [26–28]. In other words, many studies revealed that parents’ behaviors and skills, such as being role models and having good self-efficacy regarding obesity prevention in their children, play a crucial element promoting weight management among preschool children in the home environment [28–30]. However, few Iranian interventions to manage weight in Iranian preschoolers are based on behaviour change theories and models [31]. In addition, “Iran Healthy Start (IHS)/Aghazi Salem, Koodake Irani”, the customized Iranian version of “Canadian Healthy Start/Départ Santé (HSDS)”, has been developed at Mashhad University of Medical Sciences, Iran, to improve healthy eating and physical activity among preschoolers; however, there are no results until now [32].

Lifestyle is shaped during childhood and encouraging healthy behavior change is more convenient in preschool-age children (3–5 years) than in their late childhood (after age five) [4, 33]. The overall aim of this study, therefore, was to design and evaluate a behaviour change intervention programme to promote weight management in preschool children of Tehran with any weight status.

## Methods

### Theoretical framework

**Fig. 1:**
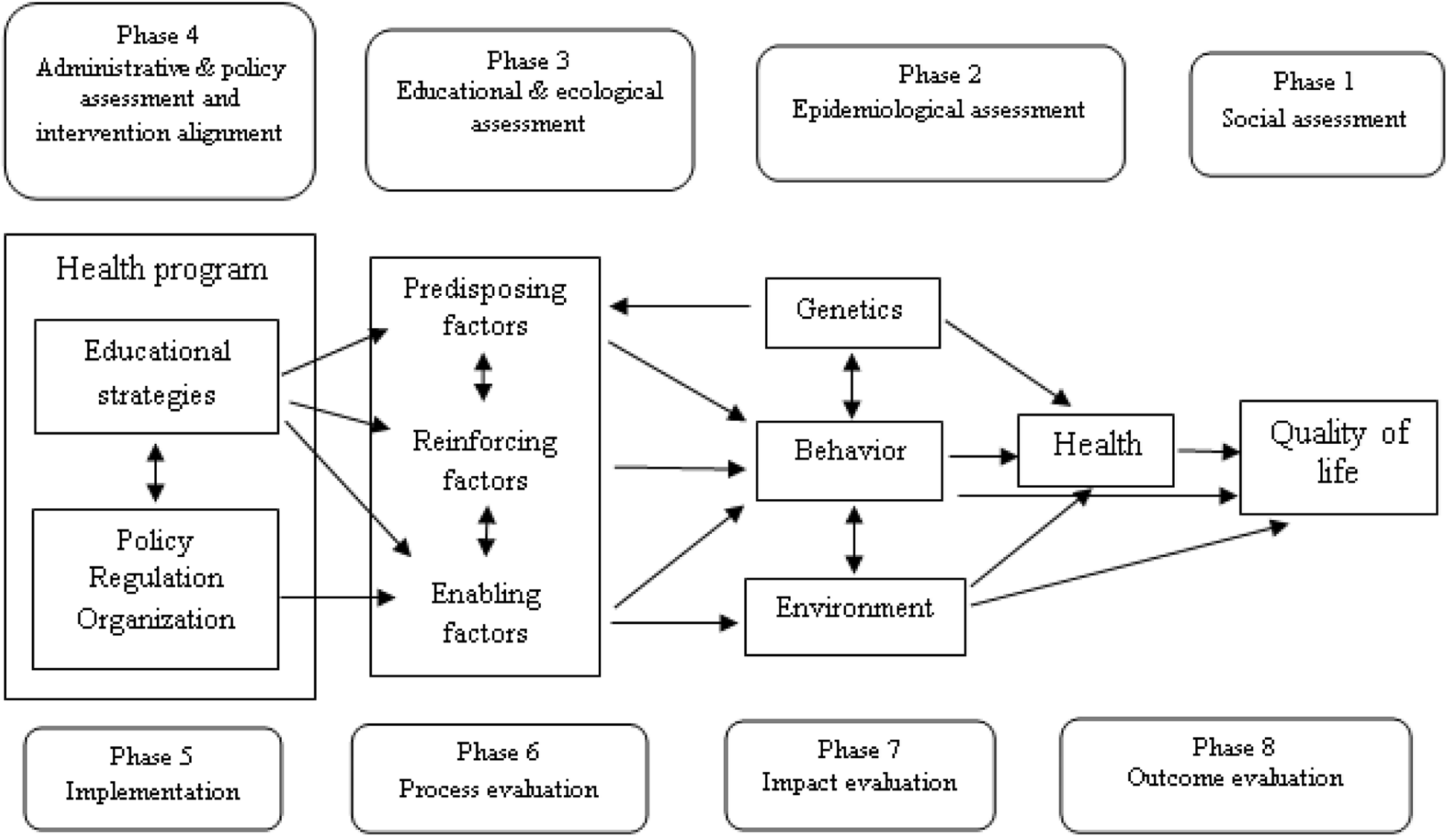
The PRECEDE-PROCEED Model

Designing and improving intervention programs need planning models as a guideline [34]. The PRECEDE-PROCEED Model (PPM), a useful and practical planning model designed by Green & Kreuter, contains 8 phases (Fig. 1) [35]. The PRECEDE (Predisposing, Reinforcing and Enabling Constructs in Educational Diagnosis and Evaluation) phase helps researchers create an outline for designing an intervention based on the distal outcomes such as quality of life [35]. Predisposing factors consist of individuals’ awareness, attitude, values, beliefs, and perceptions that facilitate or inhibit motivation for change [35]. Enabling factors, such as availability, accessibility, laws, skills, and community resources, are identified as readiness for behavioral and environmental change [35]. Reinforcing factors provide rewards or feedback for adopting and maintaining a particular behavior (such as reinforcement by health care staff, family members, teachers, peers, and community leaders) [35]. The PROCEED (Policy, Regularity and Organizational Constructs for Educational and Environmental Development) phase helps implement and evaluate the intervention developed in the first phase [35, 36]. This model is one of the most popular models in health education used to develop and evaluate most educational interventions [35]. Therefore, in this study, phase 4, 5, 7 and 8 of the model as a guideline were used for designing, implementation and evaluating the impact and outcome of a pilot study.

Electronic health (eHealth) methods in weight management interventions are more convenient and can reach larger groups than using traditional methods [37]. In addition, the increased access to technology, and smartphone applications and their advantages, such as less time for implementation, communication with different people and transmission of information create new opportunities for weight management interventions [38, 39]. Besides, social media platforms could be a good strategy for educational interventions during the COVID-19 pandemic and lockdown [40].

### Study design

This study was part of an exploratory sequential mixed methods design (qualitative-quantitative) in which a qualitative study was followed by a quantitative study and the first phase’s results were used in the second phase [41]. In the qualitative study, data were gathered through individual semi-structured interviews with open-ended questions and analyzed by using a directed content analysis approach. The study’s design and results have been described in more detail in the qualitative article [42]. Both study phases (qualitative and quantitative) have been designed and performed based on the PPM. Research indicates that SCT particularly its central component, self-efficacy, in mothers might make an impact on their children’s health lifestyle behaviors [43]. The current study was therefore designed based on a combination of PPM and self-efficacy construct to increase maternal self-efficacy to overcome barriers changing their children’s lifestyle and improvement their children’s physical activity, healthy nutrition and reducing sedentary behaviors. In this pilot study, a one-group pre and post-design was used.

The study was performed in two steps:

- 1) Design of a behavior change intervention program for weight management promotion in Tehranian preschoolers based on self-efficacy via eHealth.
- 2) Implementation and evaluation of the behavior change intervention program on Tehranian preschoolers’ dietary and physical activity and sedentary behaviors, health-related quality of life and also Body Mass Index (BMI) changes.

## Step 1: Designing a behaviour change intervention programme

### Needs assessment

Educational needs were determined by the results of the qualitative phase. The results showed that preschoolers’ screen-based behaviors, mothers’ positive perception towards having obese children, parental eating and sedentary behaviors, parents’ skills to promote children’s lifestyle and family support of children’s physical activity were recognized by participants to relate to weight management in preschoolers. The quality of parents-child relationship was also explored as a new code that affected children’s eating and physical activity behaviors. Therefore, objectives and the educational intervention were designed according to these results and a similar study conducted in Belgium [44].

According to the results of the qualitative phase, mothers played a crucial role in weight management among their children in the home environment. Mothers’ knowledge seemed a predisposing factor of the model for weight management and should consequently be improved. Attitude, as another predisposing factor, also needed to improve among mothers. These predisposing factors are precedent to behavior that motivates to change the behavior. Besides, parental eating and physical activity behaviors affected their children’s dietary and physical activity behaviors as a reinforcing factor of the model, so training mothers to be good role models was adopted. Promoting mothers’ skills to appropriately react to children’s demand for unhealthy food or sedentary behaviors was considered an enabling factor of the model. In addition, planning models, including theories and models, guided us in choosing an appropriate theory to perform the intervention programs [34]. Therefore, according to the factors identified in phase 3 of the model, self-efficacy and appropriate strategies were chosen for the intervention program in phase 4. Therefore, the objectives of the educational program were: increasing maternal self-efficacy, improving their attitude and being good role models for their children.

The researcher established a group on WhatsApp to send text messages with related pictures and provide space for discussion and asking questions for mothers to deliver educational content. In addition, a channel was made on WhatsApp to send educational videos. The educational videos are adapted from the original Dutch videos on the “www.gezondopvoeden.ugent.be” site [44], considering unique conditions in Iranian culture. Videos were watched by the research team and two mothers, and they confirmed that the videos were suitable to use for the educational intervention in Iran. Furthermore, the researcher created Persian subtitles through Subtitle Edit and WinX HD Video Converter Delux software. During the first week of the intervention, videos focused on parental skills to promote drinking water instead of soft drinks in children (2 videos); in the second week, videos focused on the consumption of fruit (2 videos). In the third week, videos included eating vegetables (2 videos); during week four, videos were sent concerning limiting sedentary behaviors (2 videos), and in the fifth week, (3) videos on the promotion of physical activity were sent. Additionally, mothers received information regarding the quality of parents-child relationship and its’ effect on their preschoolers’ lifestyle. The educational content of the program is shown in Table 1.

**Table 1:** The content and objectives of the educational program

The content of the educational program	The educational objectives	The educational method	The educational Time
Establishment a group on WhatsApp and statement the adjectives and rules	To invite and motivate mothers to participate in the study	Virtual	The first day
Asking mothers to write the below sentence "being obesity, underweight or healthy and happy of my child!" And put it on the refrigerator and think about it	Improvement of mothers' attitudes	Virtual and interactive	The first day
Explanations about the importance of weight management among preschoolers and the mother's misconception of their children's weight status along with sending some pictures	increasing mothers’ knowledge and improving their attitude	Virtual and interactive	The second day
Statement some tips regarding the importance of achieving healthy growth and development in children and express the purpose of establishment of the group, again	increasing mothers’ knowledge and their motivation	Virtual and interactive	The third day
explanations about the 5210 instruction (consumption of 5 servings of vegetables and fruits, 2 h or fewer of screen time, 1 h of physical activity and not consuming soft drinks per day) and sending relevant pictures	improvement in mothers’ knowledge	Virtual and interactive	The fourth day
Sending text messages and two educational videos about consuming enough water and no or less consuming soft drink	Improving mothers’ knowledge and skills	Virtual and interactive	Fifth to eighth days
Sending text messages and two educational videos for eating healthy snacks, including fruits	Improving mother's self-efficacy in creating appropriate rules for children	Virtual and interactive	Days ten to thirteen
Description of the food pyramid and the types of food groups	improvement in mothers’ knowledge	Virtual and interactive	Fourteenth and fifteenth days
Sending messages and two educational videos to have the healthy snacks, including vegetables	Improving the mother's self-efficacy in creating appropriate rules for children	Virtual and interactive	Sixteenth to nineteenth days
To explain the risk factors of children’s overweight and obesity (reminder)	increasing mothers’ knowledge and sensitization	Virtual and interactive	The twentieth day
Training on sedentary behaviors and sending educational videos	Improving the mother's self-efficacy in creating appropriate rules for children	Virtual and interactive	Twenty-first to twenty-sixth days
They were asking mothers to write down two sedentary behaviors that the children have done less since the past week and activities that have been replaced	Improving mothers’ self-efficacy	Virtual and interactive	Twenty-seventh day
Sending text messages about physical activity and related educational videos	Increasing the mother's skills and communication with the children, effectively	Virtual and interactive	Twenty-ninth to thirty-fifth days
Training regarding the quality of the parents-children relationship, sending two related pictures to compare and discuss with mothers	Improvement of the quality of parent–child communication	Virtual and interactive	Thirty-sixth day

## Step 2: The implementation and evaluation of the behaviour change intervention programme

### Participants and setting

Participants were mothers of 3 to 5-year-old children who attended preschool for at least four days in a week. In addition, mothers had to have a personal cell phone and the ability to send messages and be willing to participate in the study. The exclusion criteria were taking medication, practitioners’ recommendations for weight management in children, and mothers’ unwillingness to continue the study.

**Fig. 2:**
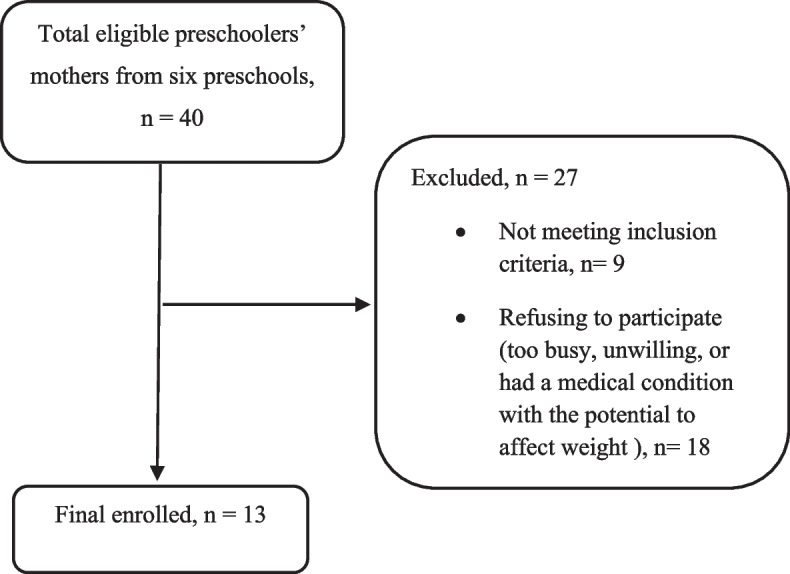
Flow diagram of participants recruitment

Phone numbers of mothers from 6 preschools in Tehran, the capital of Iran, were received. Participants were enrolled in August 2020, and after getting their permission and consent, the researcher established a group on WhatsApp, a well-known application among Iranian people. The researcher explained the objectives and methodology of the study and invited them to participate. Among 40 eligible mothers invited to the study, a convenience sample including 13 mother–child dyads (13 mothers, 14 children; one family had twins) signed the informed consent form and sent it through WhatsApp. Twenty-seven participants were excluded due to being too busy, being unwilling to participate, or having a medical condition with the potential to affect weight and not meeting inclusion criteria. Figure 2 depicts participants recruitment.

### Data collection

This study was performed between August and December 2020. At baseline, questionnaires included FFQ [45–47], the Persian version of children's health-related quality of life [48], and “the children’s physical activity and sedentary behaviors checklist” designed by researchers. An expert panel of six health education and sport sciences specialists evaluated the checklist and confirmed its content validity (CVR = 0.99). As well as demographic characteristics of parents and their preschoolers were completed by mothers. Additionally, mothers were asked to measure their child’s height and weight. Mothers received the instruction for height and weight measurements, such as removing bulky clothes and shoes before the measurements of the child’s height and weight. Based on the obtained results from children’s height and weight, the researcher assessed BMI-for-age-and-sex z-score pre- and post-test. At baseline, demographic characteristics, including sex, age, height and weight of the child, age, level of education, and parents’ occupation were received using the self-administered questionnaires. The intervention program lasted six weeks and was performed via WhatsApp. Immediately and three months after the intervention, the same measurements were performed. Additionally, at the first follow-up, mothers were asked to answer some questions about the videos. The questions were, “how did you perceive the videos?” “Were the videos interesting?” “Were the videos practical for you?” “Are you able to use the strategies” The questions were measured with a Likert scale (ranging from 5 points for completely useful, completely interesting, completely practical and yes to 1 point for completely useless, completely not interesting, completely impractical and no). Finally there was an open question: “please tell us any comments about the videos”.

The FFQ was designed for the Tehran Lipid and Glucose Study (TLGS), including 168 food items, to gain information on individuals' frequency of diet over one year. The mean reliability coefficients based on the mean energy ranged from 0.48 in people 35 years or younger to 0.65 in people over 35 years [45–47].

The Persian version of children's health-related quality of life included 22 questions in the domains of child mental health (5 items), child self-satisfaction (3 items), child movement status (3 items), child performance (4 items), parental concern regarding their children’s emotional and physical health (2 items), parental limitation (2 items), and child’s general health (3 items). The questions were scored based on a 5-point Likert scale (5: never, to 1: always, for questions 1–5 and 18–20, 5: completely satisfied to 1: completely unsatisfied for items 6–8, 5: completely false to 1: completely true for questions 14 and 15, 5: very little to1: very much for items 16 and 17, 5: excellent to 1″ poor for questions 21 and 22. Also, 4: not limited to 1: completely limited for questions 9–13). A higher score in the items of parental concern and parental limitation meant less concern and limitation, and in other items it indicates a better situation. Also, a high score on the whole child health questionnaire meant a better health status of the child. The internal consistency of the questionnaire was 0.68—0.85 and was considered moderate to high [48].

The researchers designed the children’s physical activity and sedentary behaviors checklist included eight questions. There were six questions regarding the number of days and minutes spent watching TV, using a computer and sitting games (sedentary behaviors). The questions were “How many days did your child watch TV during the past seven days” “How many minutes/day did your child watch TV”; “How many days did your child use the computer to play games during the past seven days”; “How many minutes/day did your child use the computer”; “How many days did your child play sitting games such as painting, playing with Lego, Atal Matal (a kind of sitting game among Iranian children) during the past seven days”; “How many minutes/day did your child play sitting games”. Two questions represented children's physical activities: “How many days did your child play activities, such as riding a bike, running, helping in chores, etc. during the past seven days”; “How many minutes/day did your child play activities, such as riding a bike, running, helping in chores, etc.” In the sedentary behaviors section, response: seven days received one and response: don’t know received five points, respectively. And in the minutes/day part, response: more than two hours received one and response: don’t know received six points, respectively. In the physical activity section, response: seven days received five and response: don’t know received one point, respectively. And in minutes/day part, response: more than two hours received six and response: don’t know received one point, respectively.” Spending higher days or minutes on sitting activities indicated more sedentary activities, and spending higher days or minutes on physical activity showed more activity.

### Outcome measures

In this study, changes in children’s BMI were considered the primary outcome, whereas the secondary outcomes were changes in children's health-related quality of life, dietary, physical activity and sedentary behaviors in preschool children. Based on phases seven and eight of the PPM, the outcome evaluation included assessing the weight management promotion strategies’ influence on BMI and children’s health-related quality of life. The impact evaluation involved their effect on dietary, sedentary and physical activity behaviors. All variables were measured at baseline, immediately after the intervention and three months later.

### Data analysis

Data analysis was performed using SPSS IBM statistics version 22, and the P-value of equal to and less than 0.05 was considered significant. Means, standard deviations or percentages were used according to the type of study variables. Due to the small sample size and abnormal data distribution, a Friedman test was used to evaluate changes over time.

## Results

Table 2 displays the demographic characteristics of the participants at baseline. The mean age of children was 4.19 ± 0.63, and mothers were 37.85 ± 3.13 years old. More than half of the children (57.1%) were female. The results showed that 57.1% of mothers had a bachelor's degree, and 57.2% of fathers had a master’s or Ph.D. degree. More than 85% of the mothers who participated in the study were employed.

**Table 2:** The basic demographic characteristics of the participants

Variables		% or Mean ± SD
**Children**		
Age		4.19 ± 0.63
Weight		17.65 ± 2.27
Height		105/18 ± 9.21
Sex, n (%)		
Female		8(57.1%)
Male		6(42.9%)
**Parents**		
Age of mothers		37.85 ± 3.13
Age of fathers		41.28 ± 4.28
Educational status, n (%)		
Mother	Diploma	0(0%)
	Associate in Science	0(0%)
	Bachelor of Science	8(57.1%)
	Master of Science	5(35.7%)
	Doctor of philosophy	1(7.1%)
Father	Diploma	2(33.3%)
	Associate in Science	1(7.1%)
	Bachelor of Science	3(21.4%)
	Master of Science	4(28.6%)
	Doctor of philosophy	4(28.6%)
Employment status, n (%)		
Mother	Housewife	2(14.3%)
	employed	12(85.7%)
Father	employed	100%

Table 3 shows the BMI z-score status in preschoolers at baseline, immediately after the intervention and three months later.

**Table 3:** BMI z-score status in preschoolers at baseline, immediately after the intervention and three months later

BMI z-score	Baseline	immediately	Three months
normal-weight	12(85.7%)	13(92.8%)	12(85.7%)
at risk of being overweight	1(7.1%)	-	-
overweight	-	-	1(7.1%)
obesity	1(7.1%)	1(7.1%)	1(7.1%)
Total	14	14	14

Three mothers out of 13 mothers reported watching all of the educational videos, and two mothers did not watch the videos. Among 11 mothers, nine (81.8%) stated that the videos were practical, and eight mothers (72.7%) found them useful. There was only one mother who suggested adapting the videos for Iranian people.

The baseline, immediately and three months after the intervention, values for BMI z-score, FFQ scores, the Persian version of children's health-related quality of life items and the children’s physical activity and sedentary behaviors checklist are reported in Table 4. BMI z-score was 2.29 ± 0.82, 2.21 ± 0.80 and 2.35 ± 0.92, respectively, at baseline, immediately and three months after the intervention with no statistically significant difference (*P* = 0.6).

**Table 4:** Effects of educational intervention on BMI z-score, FFQ, the Persian version of children's health-related quality of life, and the duration of physical activity and sedentary behaviors checklist

Variables	Baseline Mean ± SD	Immediately Mean ± SD	Followup Mean ± SD	χ^۲ *^	*p*-value
**BMI z-score**	2.29 ± 0.82	2.21 ± 0.80	2.35 ± 0.92	1.00	0.60
**FFQ**					
Calories	2537.48 ± 761.59	2188.62 ± 802.32	1864.47 ± 903.18	5.53	0.06
Whole grains	32.95 ± 14.52	45.12 ± 42.99	50.40 ± 31.62	5.32	0.07
Refined grains	257.39 ± 73.71	278.70 ± 55.23	261.69 ± 112.67	1.24	0.53
Meats	99.39 ± 58.98	81.61 ± 40.05	97.23 ± 39.23	2.62	0.26
Dairy products	732.70 ± 346.80	737.19 ± 524.64	543.81 ± 287.20	2.94	0.23
Legumes	36.47 ± 22.53	33.21 ± 17.38	43.10 ± 20.85	1.21	0.54
Fruits	997.60 ± 680.25	568.29 ± 240.68	515.70 ± 324.59	8.98	0.01^**^
Vegetables	259.72 ± 96.21	236.85 ± 105.02	228.67 ± 125.74	0.03	0.98
Liquid fats	9.78 ± 4.67	7.95 ± 3.56	9.32 ± 4.07	2.85	0.24
Solid oils	13.00 ± 16.66	8.69 ± 12.63	6.79 ± 7.04	0.93	0.62
Nuts and seeds	19.38 ± 25.39	14.99 ± 21.59	10.70 ± 15.72	0.00	1.00
Tea and coffee	106.29 ± 102.97	142.33 ± 106.60	138.23 ± 105.48	2.31	0.31
Salty snacks	4.62 ± 7.47	1.74 ± 2.22	2.93 ± 5.42	3.24	0.19
Simple sugars	4.20 ± 4.81	5.02 ± 4.87	5.68 ± 4.52	6.54	0.03^**^
Honey and jams	7.44 ± 6.12	7.16 ± 5.01	8.43 ± 7.69	0.57	0.74
Soft drinks	260.79 ± 211.19	145.87 ± 136.38	93.77 ± 65.50	14.08	0.001^**^
Snacks and desserts	43.13 ± 28.42	36.80 ± 26.15	48.91 ± 37.91	5.57	0.06
Fast food	20.86 ± 11.84	39.46 ± 32.98	34.63 ± 29.26	3.64	0.16
others	6.73 ± 5.81	5.26 ± 3.38	7.40 ± 5.57	1.75	0.41
**The Persian version of children's health-related quality of life**					
child mental health	18.50 ± 3.13	18.64 ± 3.73	17.57 ± 2.65	3.56	0.16
child self-satisfaction	12.07 ± 1.73	11.64 ± 1.33	11.92 ± 1.85	0.31	0.85
child movement status	10.57 ± 2.31	10.64 ± 2.13	11.85 ± 0.53	3.63	0.16
child performance	16.50 ± 1.99	16.57 ± 2.20	17.28 ± 1.13	2.05	0.35
parental concern	7.50 ± 2.47	8.71 ± 1.58	8.71 ± 1.54	12.21	0.002^**^
parental limitation	7.92 ± 1.85	8.64 ± 1.39	8.42 ± 1.45	3.00	0.22
parental perception of their children’s general health	11.28 ± 1.81	12.0 ± 2.14	12.42 ± 2.27	5.90	0.05^**^
child health questionnaire	84.35 ± 12.83	86.85 ± 11.56	88.21 ± 8.16	2.73	0.25
**The duration of physical activity and sedentary behaviors checklist**					
days spent for watching TV during a week	5.78 ± 1.47	6.57 ± 0.85	6.24 ± 0.93	4.33	0.11
minutes spent for watching TV during a day	65.35 ± 33.46	76.60 ± 40.88	84.64 ± 36.43	3.81	0.14
days spent using computer during a week	2.50 ± 2.73	3.50 ± 2.65	2.78 ± 2.83	0.66	0.71
minutes spent for using computer during a day	25.71 ± 27.70	24.10 ± 16.42	30.53 ± 31.33	0.16	0.92
days spent sitting games during a week	5.85 ± 1.83	6.0 ± 1.03	5.85 ± 1.02	0.22	0.89
minutes spent for sitting games during a day	56.42 ± 37.97	65.89 ± 37.57	73.39 ± 31.15	4.15	0.12
days spent physical activities during a week	4.35 ± 2.70	5.35 ± 1.90	4.85 ± 2.07	2.43	0.29
minutes spent for physical activities during a day	36.42 ± 32.02	58.03 ± 36.88	53.75 ± 30.12	7.53	0.02^**^

*SD* Standard Deviation

^*^Friedman Test

^**^ *P*-value ≤ 0/05

At baseline, immediately and three months after the intervention, the minutes that children were involved in physical activity per day increased significantly (*P* = 0.02). At first follow-up, children were more active compared to baseline and three months after the intervention.

The result of the Friedman test showed an increase and significant difference in the parental concern regarding their children’s emotional and physical health between baseline and immediately after the intervention (*P* = 0.002); however, the score did not show much difference at three months after the intervention compared to immediately after the intervention. The parental perception of their children’s general health increased significantly (P = 0.05). No significant differences were observed in the other items of the Persian version of children's health-related quality of life between baseline, immediately and three months after the intervention.

At baseline, immediately and three months after the intervention soft drink consumption reduced significantly (*P* = 0.001). However, fruit intake decreased significantly (*P* = 0.01), and simple sugars such as cube sugar and candy increased significantly (*P* = 0.03).

## Discussion

In this pilot study, we found no significant difference between the BMI z-scores before and after the intervention. There were, however, significant increases in physical activity, some components of children’s health-related quality of life, and simple sugar intake. We observed a significant reduction in sugar-sweetened beverage and fruit consumption after the 3-month intervention.

Since BMI normally increases as part of the normal growth and development process among children aged 3–5, the fact that the BMI stayed stable in this study can be considered a positive outcome. Findings of studies on weight management among preschoolers reported various results including no to significant intervention effects on BMI [16, 49]. In addition, the results of a meta-analysis demonstrated that interventions targeting overweight and obese children were more effective than interventions targeting both non-overweight/obese and obese-overweight children [50]. In the current study, at baseline, 85% of children were normal-weight. Another reason for not seeing any significant changes in BMI could be that other mediators are more crucial for changing BMI, such as parenting style, family functioning and socio-economic situation, which might be examined in future studies.

Other interventional studies have demonstrated similar improvements in physical activity among preschoolers [51, 52]. In addition, a systematic review and meta-analysis reported that educational interventions based on social media sites resulted in an increase in physical activity among children aged 6–18 years [53]. Even though our intervention was performed during the COVID-19 pandemic and people had to stay at home, there was a cyclical lockdown and individuals could go out and be physically active within some restrictions (e.g., social distancing and wearing a mask). Increased physical activity might, furthermore, be due to suggested activities and intervention strategies utilized in this study for children. For example, mothers were suggested to let their children build caves and labyrinths out of blankets, chairs, and tables and move between them and to use stairs instead of elevators with their children.

A decline in sugar-sweetened beverage consumption was also reported in a preschoolers’ parent-focused eHealth intervention, similar to our study [16]. A body of evidence showed that multiple intervention components did not report much success in terms of BMI. However, some have revealed improvement in other components, such as reduction in sugar-sweetened beverage intake [54, 55] and an increase in water intake [50, 53]. Some studies reported that sugar-sweetened beverage intake was related to cultural norms, parents’ habits of utilizing soft drinks and their availability at home [56, 57]. Therefore, this reduction may be positive parental modeling as a moderator to decrease sugar-sweetened beverage consumption.

A body of evidence showed that multidisciplinary intervention programs among overweight and obese children aged 3–18 improved their quality of life [58, 59]. The results of two systematic reviews reported that increased physical activity levels resulted in a better quality of life among children and adolescents aged 3–18 years [60, 61]. Hence, the improvement in quality of life among preschoolers in the present study may be due to the increase in physical activity behavior.

Our study results regarding children’s reduced fruit consumption after three months were contrary to previous eHealth studies that demonstrated an increase in fruit intake among preschoolers and adolescents [62, 63]. Studies indicated that fruit consumption was directly or indirectly dependent on the fruit availability at home [64, 65].

In contrast to increasing simple sugars intake, such as cube sugar and candy in our study, Esfarjani et al. reported a decrease in sugar and confectionary ingredients’ intake among seven-year-old Iranian obese children through a family-based intervention [66]. However, Hammersley et al. did not observe a significant change in sugar intake in an Australian study using an internet-based childhood obesity prevention program for parents [16]. An explanation could be that the intervention focused on multiple behaviors instead of one behavior. In addition, Iranian people tend to bake cookies and cakes for their children and guests. Therefore, the likely reason that can be put forward for the high level of sugar intake among the participants may be cooking these kind of foods, especially during their quarantine time in the COVID-19 pandemic. As reported by Jansen et al., children aged 2–12 years had sweeter snacks due to COVID-19-specific stress with potential influence on their obesity risk [67].

### Strengths and limitation

There are several limitations of this study: self-reporting of data may include reporting bias, which makes spurious relationships between the measure being reported and factors affecting reporting. It may overestimate physical activity levels and underestimate children’s anthropometric measurements, thus their BMI. Besides, the lack of a control group threatened the internal validity. In addition, the sample size of this study was not powered to detect statistically significant differences in some outcomes, such as food items. Another limitation was that the study did not measure mothers’ self-efficacy. Future studies, therefore, should examine the impact of childhood obesity prevention programs on maternal outcomes, such as mothers’ self-efficacy and skills.

According to behavior change theory, using eHealth to transfer strategies for behavior change improvement in the early years, the use of an intervention should be considered a strength of this study.

## Conclusion

Preschoolers depend on their parents to provide healthy food, so parents have a crucial role in preschooler’s healthy lifestyle. Using a theory-based educational intervention for Iranian mothers, delivered via social media sites, successfully produced some children’s behavior changes related to diet, physical activity, and quality of life. Although, results from this pilot study should be interpreted with caution and should be replicated on a larger scale to evaluate whether effects are maintained in a larger sample.  

## Data Availability

The datasets generated and/or analysed during the current study are not publicly available due to anonymity of individuals who attended in the study, but are available from the corresponding author on reasonable request.

## Abbreviations

BMI: Body Mass Index
FFQ: Food Frequency Questionnaire
HBM: Health Belief Model
HSDS: Canadian Healthy Start/Départ Santé
IHS: Iran Healthy Start
PPM: PRECEDE-PROCEED Model
TPB: Theory of Planned Behavior
SCT: Social Cognitive Theory
TLGS: Tehran Lipid and Glucose Study
WHO: World Health Organization
